# Effects of asymmetric load bench press offset training on muscle activation levels and exercise-induced fatigue in collegiate bodybuilders

**DOI:** 10.3389/fphys.2025.1592477

**Published:** 2025-05-09

**Authors:** Bin Yan, Siqi Yao, Junjie Zhang, Chunwei Li, Tongxin Han, Qiushi Hu, Kai Lv

**Affiliations:** ^1^ Academic Affairs Office, Henan Sports University, Zhengzhou, Henan, China; ^2^ School of Physical Education, Zhengzhou University (Main Campus), Zhengzhou, Henan, China; ^3^ Kangda College, Nanjing Medical University, Nanjing, Jiangsu, China; ^4^ Department of Physical Education, Tianjin Medical University, Tianjin, China

**Keywords:** interlimb asymmetry, electromyography, resistance training, neuromuscular adaptation, fatigue threshold, core compensation

## Abstract

**Objective:**

This study systematically investigated the effects of graded asymmetric load bench press offset training on muscle activation patterns, exercise-induced fatigue, and movement performance in bodybuilders.

**Methods:**

Ten male athletes (age: 24.20 ± 1.91 years; 1 R M bench press: 120.00 ± 14.66 kg) underwent randomized crossover trials with 0% (symmetrical), 2%, 4%, and 6% asymmetric load interventions (70% 1 R M total load). Surface electromyography (sEMG) quantified activation levels of pectoralis major (PM), anterior deltoid (AD), triceps brachii (TB), and external oblique (EO), while barbell kinematics, blood lactate, and heart rate were analyzed to assess fatigue.

**Result:**

Key findings revealed significant interlimb asymmetry under symmetrical loading, with dominant-side PM (51 ± 6.82 vs 35 ± 5.32 MVIC%, p = 0.009) and AD (48.2 ± 5.05 vs 32.6 ± 9.21 MVIC%, p = 0.038) exhibiting higher activation than the non-dominant side. Asymmetric loading effectively mitigated this imbalance: 6% intervention increased non-dominant PM (54.4% ± 8.46% vs 0%: 35 ± 5.32 MVIC%, p = 0.035) and AD activation (52.3% ± 12.7% vs 0%: 32.6 ± 9.21 MVIC%, p = 0.022), but triggered compensatory EO recruitment (31.1% ± 12.3% vs 0%: 12.8 ± 3.34 MVIC%, p < 0.001). Performance metrics declined progressively with higher asymmetry: 6% loading reduced barbell velocity (MV: 0.28% ± 0.03% vs 0%: 0.38 ± 0.04 m/s, p < 0.001), repetitions (6.63% ± 2.40% vs 0%: 13.90 ± 2.52, p < 0.001), and power (MP: 357% ± 43% vs 0%: 437 ± 53.70 W, p = 0.009). Physiological fatigue markers intensified at 6% asymmetry, evidenced by elevated post-exercise blood lactate (7.42% ± 1.59% vs 0%: 9.88 ± 0.75 mmol/L, p = 0.003) and prolonged heart rate recovery.

**Conclusion:**

The study identifies 2%–4% asymmetric loading as optimal for enhancing non-dominant muscle activation while minimizing fatigue, whereas 6% interventions induce core compensation and performance deterioration. These findings establish evidence-based thresholds for precision training protocols, addressing interlimb asymmetry while balancing neuromuscular efficacy and physiological strain. Methodological innovations include multidimensional analysis of biomechanical, electromyographic, and physiological responses, advancing the understanding of neuromuscular coordination in asymmetric resistance training.

## 1 Introduction

The bench press (BP), a comprehensive exercise involving multiple joints and muscle groups, is the preferred training method for athletes, bodybuilders, and weightlifters aiming to enhance upper limb strength and explosiveness (Elliott et al., 1989). Research has demonstrated inherent interlimb asymmetries and biases in the human body, leading to a typically unstable performance of the bench press (Anderson and Behm, 2004; Maloney, 2019). Consequently, strategies to synergize core muscles, responsible for trunk stability, with upper limb muscles, which provide bench press strength, have become a focal point of recent investigations (Shinkle et al., 2012). Scholars have proposed various intervention techniques tailored to the “instability” characteristic of bench press movements. These include alterations in the supporting surface (Saeterbakken and Fimland, 2013), selection of different bench press bars (Costello, 2022), incorporation of swinging loads (either front or side swinging) (Saeterbakken et al., 2020a), and variations in supine trunk position, such as feet-on-ground or active hip and knee flexion (Muyor et al., 2019). The primary objective of these methods is to examine muscle activation patterns during the exercise, aiming to bolster the coordination between the upper limb and core muscle groups, thereby mitigating sports injuries and enhancing bench press proficiency (Costello, 2022; Saeterbakken et al., 2020b; Jarosz et al., 2020; Golas et al., 2018; Lawrence et al., 2021; Ostrowski et al., 2017). Notably, many extant studies predominantly focus on the dominant limb side, overlooking muscle activation changes on the non-dominant side. Golas et al. (2018) observed distinct muscle activation disparities between the dominant and non-dominant upper limbs post-bench press in elite athletes, with the dominant side exhibiting significantly higher activation. Thus, relying solely on data from the dominant side to assess neuromuscular patterns and muscle activation during the bench press may be inconclusive. The observed disparities in muscle activation between the dominant and non-dominant sides can be attributed to interlimb asymmetry effects. Inter-Limb Asymmetries (ILA) quantify the variances in physical attributes such as strength, explosiveness, flexibility, and balance between the left and right sides of the human body. Such asymmetry may stem from inherent factors, like limb dominance, or acquired factors, such as specialized sports training or disparate recovery rates following an injury. These asymmetries can profoundly influence athletic performance and predispose individuals to injuries (Maloney, 2019; Bishop et al., 2023). To mitigate these effects, one can employ targeted strength training, functional training, and biomechanical corrections to harmonize limb capabilities, thereby enhancing overall athletic performance and diminishing injury risks. Recent studies have highlighted the neural control mechanisms underlying these asymmetries, particularly in the upper limbs. Lecce et al. (2025) demonstrated that dominant muscles exhibit greater maximal voluntary force (MVF) and higher motor unit discharge rates, attributed to increased neural drive from greater shared synaptic inputs. This neural drive is not solely due to intrinsic motoneuron properties but is modulated by the distribution of synaptic inputs. Furthermore, cross-education phenomena, where unilateral training affects the contralateral limb, have been shown to enhance muscle activation through neural adaptations at both spinal and supraspinal levels (Lecce et al., 2024). Consequently, investigating the most effective training interventions for rectifying limb asymmetry holds considerable significance.

Offset Training, a novel training method, capitalizes on the disparity in muscle activation levels between the dominant and non-dominant sides of the body. It enhances balance and stability requirements by intentionally creating an asymmetrical distribution of external loads during bilateral resistance training. This significantly increases the activation level of the muscles on the loaded side. Offset Training is effective in rectifying muscle strength imbalances, diminishing injury risk, and enhancing overall athletic performance (Jarosz et al., 2020). The method can be applied to various forms of bilateral resistance training such as flat bench presses, squats, and deadlifts. Several scholars have employed asymmetrical load interventions during bench press exercises (Saeterbakken et al., 2020b; Jarosz et al., 2020), investigating fluctuations in muscle activation levels on the dominant and non-dominant sides. The aim is to identify sports interventions that augment the stability of the bench press. Jarosz et al. (2020) pioneered an analysis of electromyographic test results during the bench press under asymmetric load interventions. They designed comparative experiments with asymmetric load interventions of 2.5%, 5%, and 7.5% on both sides. Their findings revealed that regardless of the side (dominant or non-dominant) where the load was applied, the muscle activation level on the loaded side escalated to varying degrees. However, Saeterbakken et al. (2020b) found in subsequent research that larger asymmetric load interventions (unloading 5% and 10% on the non-dominant side) may challenge subjects to maintain the stability of the bench press movement, potentially causing barbell rod movement. Furthermore, interventions involving asymmetric loads of 5% and 10% resulted in an increase in muscle activity in the core muscle groups on the loaded side by 280% and 320%, respectively. Consequently, there were minimal alterations in the activation levels of the primary muscles in both the dominant and non-dominant upper limbs, such as the triceps brachii (TB), anterior deltoid (AD), and pectoralis major (PM). In a recent study (Sharp et al., 2022), Matthew Sharp discovered that 4 weeks of bench press offset training with an asymmetric load of 5% can enhance both muscle thickness and bench press strength more effectively than traditional symmetrical load training. This underscores the superiority of offset training for muscle hypertrophy and strength gains. It is important to note that none of the aforementioned studies addressed the issue of exercise-induced fatigue. Given the significant difference in muscle recruitment patterns between asymmetric load offset training and traditional training, excessive exercise-induced fatigue could negatively impact neuromuscular recruitment. Therefore, it is crucial to investigate the incidence of exercise-induced fatigue during asymmetric load offset training. In conclusion, this study aims to mitigate the increased instability during the bench press caused by significant asymmetrical load interventions (Saeterbakken et al., 2020b). This study builds upon the experimental design of Jarosz et al. (2020), which utilized 2.5%, 5%, and 7.5% asymmetrical load interventions, to further refine the minimum threshold at which asymmetrical load interventions significantly impact muscle activation levels. The goal is to enhance the effectiveness and cost-efficiency of these interventions. Consequently, interventions of 0% (symmetrical load), 2%, 4%, and 6% asymmetrical loads were selected. In analyzing the changes in muscle activation levels of the target upper limb muscles, the study also included the activation changes of the core muscle group’s External Oblique (EO). This comprehensive approach allows for a deeper evaluation of the muscle force characteristics of the upper limb and core muscle groups during asymmetrical load bench press offset training. By examining the changes in athletic fatigue under varying asymmetrical load offset trainings, this study aims to precisely understand the beneficial effects of different degrees of asymmetrical load interventions on the stability of the bench press movement. Based on these findings, we hypothesize that graded asymmetric load bench press training will enhance non-dominant muscle activation by increasing neural drive to these muscles. We also expect that this enhanced activation will lead to improved force generation and reduced interlimb asymmetry. Additionally, we anticipate that higher asymmetry levels may induce greater physiological fatigue.

## 2 Objective and methods

### 2.1 Objective

The study was conducted from October to November 2024 in a physical training room, employing a research design that combines randomized crossover control with self-control methods. The sample size for this study was determined using G*Power software (version 3.1). The parameters used in the analysis were as follows: an F-test for ANOVA (repeated measures, within factors), a desired power level of 0.8, and an expected effect size \(f = 0.56\), which was based on prior studies (Saeterbakken et al., 2020c)^.^ Considering a potential 20% sample loss, the initial calculated sample size was seven subjects. Ultimately, a total of 10 participants were enrolled in the study. Through strict inclusion criteria, a final group of 10 qualified subjects was formed for the study, with their basic information detailed in Table 1. To minimize the risk of subjective bias, the subjects were not informed of the true and comprehensive purpose of the experiment. Before the experiment, they were only given a general overview: that the research was related to the effects of asymmetric load bench press offset training on muscle activation levels and exercise - induced fatigue responses of the target muscle groups on the dominant and non - dominant sides. This general description was provided to ensure that the subjects could understand the basic nature of the experiment and cooperate with the procedures, while keeping them unaware of the in - depth research goals, such as precisely evaluating the optimal asymmetric load thresholds for enhancing non - dominant muscle activation and minimizing compensatory fatigue, and comprehensively understanding the long - term impacts of asymmetrical load training on muscle force characteristics and movement stability. This way, the potential influence of subjects’ subjective expectations on the experimental results was reduced. The selection of male subjects was made to control for potential influences of gender-related hormone level fluctuations and physiological cycle changes on muscle activation degrees and training effects, ensuring comparability and reliability of electromyographic signals, force output, and other physiological parameter measurements during the experiment. Previous research has shown that menstrual cycle phases can influence muscle activation patterns and neuromuscular performance due to hormonal fluctuations (Piasecki et al., 2023).

**TABLE 1:** List of basic physiological information of subjects (n = 10).

Years (age)	Height (cm)	Weight (kg)	Years of training	Bench press1RM(kg)
24.20 ± 1.91	174.10 ± 2.93	76.90 ± 6.19	4.55 ± 0.80	120.00 ± 14.66

Eligibility criteria included: (1) healthy men aged 19–24 with no chronic diseases or acute injuries; (2) a minimum of 3 years of consistent resistance training experience, with a current regular training regimen; (3) a 1 R M bench press capability of at least 120% of body mass, ensuring a robust foundation in upper extremity strength; (4) proficiency in the standard bench press technique, demonstrating precision in both eccentric and concentric phases as per the experimental protocol; and (5) no significant sports injuries within the past year and no conditions or surgical histories impacting upper limb or core muscle group functionality. The exclusion criteria were as follows: (1) Participation in high-intensity resistance training or aerobic exercise within 72 h prior to the experiment, as this could potentially influence the accuracy of the results. (2) The use of any substances or equipment that could impact muscle performance, such as stimulants, steroids, caffeine, and weightlifting belts, wrist wraps, elbow sleeves, *etc.* (3) Presence of musculoskeletal issues that could hinder bench press performance, including chronic pain in the shoulder, elbow, or back, arthritis, or other similar injuries. (4) Psychological conditions that could potentially affect performance during the experiment, such as anxiety, depression, or other psychiatric disorders.

### 2.2 Methods

#### 2.2.1 Experimental design and process

Throughout the entire experiment, participants were required to perform four sets of bench press training with different asymmetric loads. Sessions with varying degrees of asymmetric load bench press training were separated by 48-hour intervals to eliminate interference effects between sessions. The asymmetric loads in this experiment were divided into four levels: 0% asymmetry, 2% asymmetry, 4% asymmetry, and 6% asymmetry. The order of these asymmetric load conditions was randomized through participant drawing of lots to ensure randomization, parallelism, and avoidance of cumulative training effects. In all asymmetric load conditions, the total load was maintained at 70% 1 R M (accurate to 0.25 kg). However, the load distribution between the dominant and non-dominant sides differed across conditions. The dominant side was defined as the arm participants preferentially used for throwing (Saeterbakken et al., 2020b). A 1% 1 R M load served as the base value for the load difference between sides, with the dominant side unloaded and the non-dominant side loaded asymmetrically. Specific load designs were as follows:

0% asymmetry: Equal load on both dominant and non-dominant sides.2% asymmetry: Dominant side unloaded by 1% 1 R M, non-dominant side loaded by 1% 1 R M; 4% asymmetry: Dominant side unloaded by 2% 1 R M, non-dominant side loaded by 2% 1 R M; 6% asymmetry: Dominant side unloaded by 3% 1 R M, non-dominant side loaded by 3% 1 R M.The asymmetric load design was adapted from previous studies (Saeterbakken et al., 2020b; Jarosz et al., 2020) with modifications to refine the minimum threshold of asymmetric load intervention required to significantly affect muscle activation levels. This study strictly adheres to the Helsinki Declaration and has been approved by the Ethics Committee of Henan Sport University.

**FIGURE 1:**
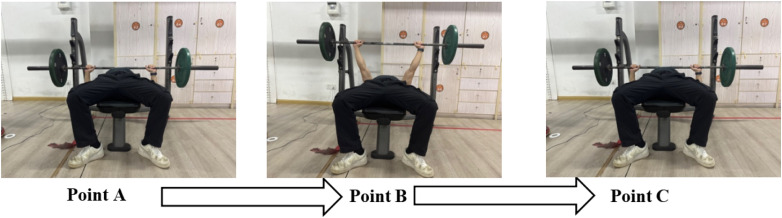
Eccentric, concentric phase of bench press.

Three days prior to the formal asymmetric load intervention bench press experiment, baseline information of subjects shall be collected and 1 R M bench press testing shall be conducted. On the experimental day, maximal voluntary isometric contraction (MVIC) measurements should first be performed on eight target muscles: the pectoralis major, triceps brachii, anterior deltoid, and external oblique muscles of both dominant and non-dominant body sides. Subsequently, subjects will perform asymmetric load bench press offset training with controlled movement rhythm and trajectory (using a metronome to maintain 2s duration for eccentric phase (Point A→B) and 1s for concentric phase (Point B→C), ensuring temporal consistency of movement execution). Throughout the exercise, subjects must maintain continuous contact of head, shoulders, and hips with the bench. The barbell must touch the chest during descent and achieve full elbow extension at the top position to ensure standardized movement execution as illustrated in Figure 1.

**FIGURE 2:**
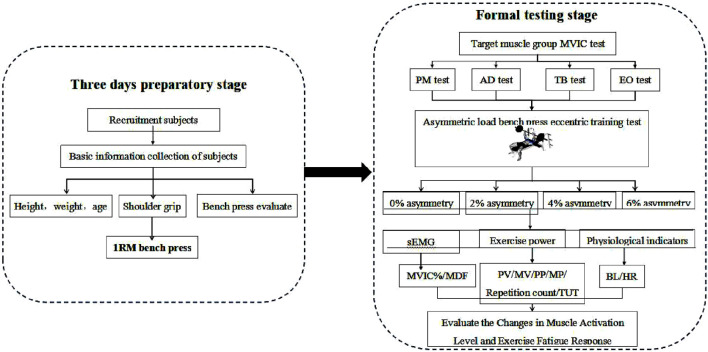
Experimental flowchart.

Each set of asymmetric load bench press training requires subjects to perform repetitions until failure. The criteria for failure determination follow previous research (Krzysztofik et al., 2021): when subjects cannot complete another concentric movement through the full range of motion during bench press, accompanied by movement deviations (e.g., barbell path deviation, shoulder/hip lift-off from the bench), inability to maintain movement rhythm, or decreased core stability, failure is confirmed, and the test is immediately terminated. Throughout the asymmetric load bench press offset training, surface electromyography (sEMG) changes of target muscles, barbell velocity/power output, as well as pre- and post-training heart rate and blood lactate levels are recorded to evaluate muscle activation patterns and exercise-induced fatigue responses under varying degrees of asymmetric loading. The detailed experimental flowchart is presented in Figure 2.

#### 2.2.2 Main test and observation indicators

**FIGURE 3:**
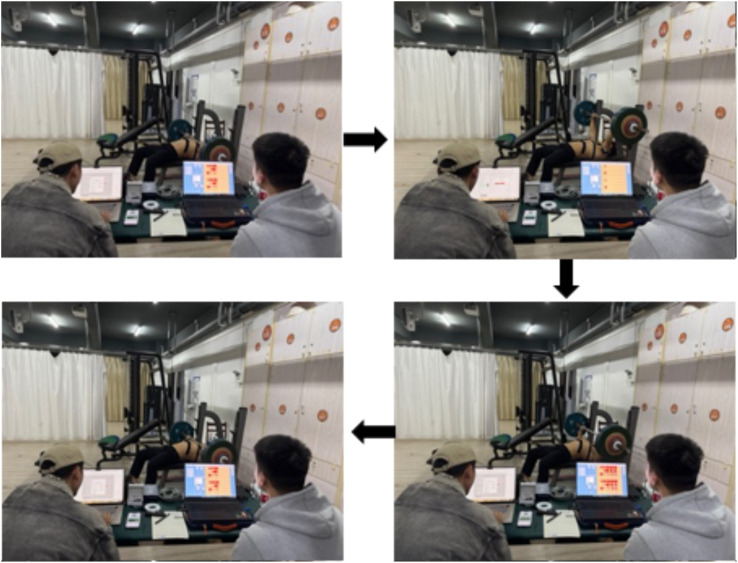
Subjects performing the bench press 1 R M test.

**FIGURE 4:**
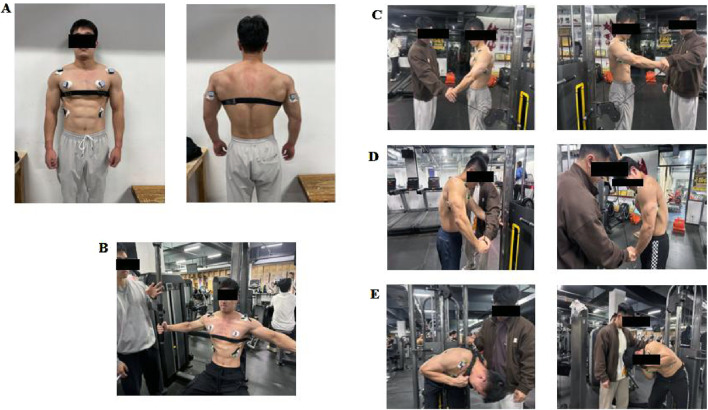
Target Muscle Surface EMG Acquisition Process **(A)** Target muscle surface EMG electrode attachment point **(B)** MVIC test of the dominant and non-dominant sides of the pectoralis major muscle **(C)** MVIC test of the dominant and non-dominant sides of the anterior deltoid muscle **(D)** MVIC test of the dominant and non-dominant sides of the triceps brachii muscle **(E)** MVIC test of the dominant and non-dominant sides of the external oblique muscle.

(1) Bench Press 1 R M Test: All participants were evaluated for their bench press 1 R M 48 h prior to the commencement of the experimental phase. This assessment determined their maximal strength levels and provided normalized loading parameters for subsequent experiments. Prior to the test, participants underwent a 10-minute standardized warm-up, which included a whole-body warm-up using a power cycle (resistance set at 100 W and cadence maintained at 70–80 rpm). This was designed to elevate heart rate and promote overall blood circulation in preparation for the high-intensity tests that followed. Subsequently, participants performed specific warm-up sets of 15, 10, and five repetitions with loads of 20%, 40%, and 60% of their verbally reported estimated 1 R M, respectively. This progression was intended to activate relevant muscle groups incrementally and familiarize participants with the bench press movement pattern. For the formal test, subjects commenced with an 80% load of 1 R M, performing three to five repetitions. Subsequent attempts increased by 4–9 kg. A 3-min rest interval was enforced between sets to replenish energy levels. The weight was then incrementally increased by 4–9 kg with two to three repetitions. Upon achieving a successful 1 R M, the weight continued to escalate by 4–9 kg. If unsuccessful, the weight decreased by 2–4 kg. The 1 R M was ascertained from three to five attempts and the protocol was repeated until the subject could no longer complete the lift. To ensure both the efficiency of the testing and the subject’s safety, all subjects’ 1 R M were determined within five attempts. Throughout the test, subjects were mandated to adhere strictly to the standardized technical bench press requirements. This included allowing the barbell to descend to the chest, pushing it upwards until the elbows were fully extended, and maintaining contact of the head, shoulders, and buttocks with the bench during the entire movement. This discipline was essential for the accuracy and reliability of the test results. The detailed procedure is illustrated in Figure 3.
(2) MVIC test of the target muscle group: Prior to the formal test, muscle electromyographic values were recorded under maximal voluntary isometric contractions 5 minutes earlier. This was done to normalize the surface integral electromyographic values in line with SENIAM procedures (Hermens et al., 2000). Electrodes were placed on four muscles bilaterally: the trapezius descendens (TB), anterior deltoid (AD), triceps brachii (PM), and external oblique (EO). The skin covering the muscle belly was shaved and cleaned with alcohol to prepare for the placement of gel-coated, self-adhesive electrodes. For the pectoralis major, electrodes were positioned 4 cm medially from the axilla on the costal fibers; for the anterior deltoid, 1.5 cm anteriorly from the acromion process; for the triceps brachii, medial and inferior to the long head belly; and for the external oblique, on the abdominal external oblique muscle belly. Figure 4A illustrates the subjects’ target muscle electromyography motor attachment points. During each MVIC trial, participants were instructed to gradually increase force production, achieving their perceived maximum force within 3 s, and then sustain maximal effort for a duration of 3–5 s. Following this, they were to slowly release the muscle tension over a 3-s period, subsequently transitioning gradually back to a resting state. Each muscle was subjected to two trials, separated by a 1-min rest period. The precise testing procedures for each target muscle’s MVIC are delineated below.

**FIGURE 5:**
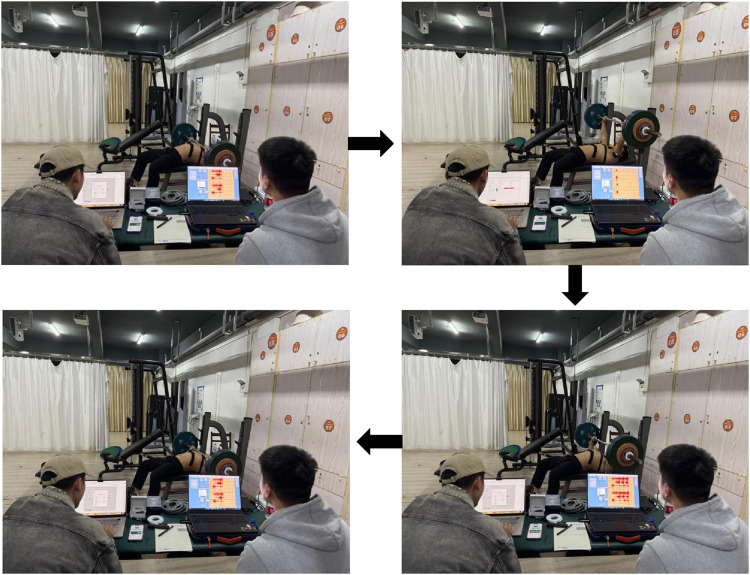
Surface EMG and motor power acquisition process during offset training with asymmetric load bench press.

① Method of measuring pectoralis major MVIC: The subject sits upright on a butterfly chest press machine, with their chest lifted and abdomen tightened, arms slightly bent, and performs a maximum effort chest press at an elbow angle of 170° under maximum load, holding for 3–5 s. The electromyographic data of the pectoralis major muscle is collected at this time.
② The measurement method of the anterior deltoid muscle MVIC: The subject stands sideways in front of the gantry, holding a steel wire to perform a front raise, maintaining a 120° angle between the upper arm and forearm. Then, the subject exerts full effort to raise it upwards, while the tester applies downward resistance, persisting for 3–5 s, capturing the electromyographic data of the anterior deltoid muscle at this moment.
③ Method of measuring the MVIC of the triceps brachii muscle (long head): The subject performs the dragon-gate steel wire arm flexion and extension movement in a forward-leaning position, maintaining a 120° angle between the upper arm and the forearm. The upper arm is clamped to the body and does not move, then the subject exerts full force to extend the forearm while the tester applies a counterforce from the side rear. This position is held for 3–5 s, and the electromyographic data of the triceps brachii muscle is collected at this time.
④ Measurement method for external oblique abdominal muscle MVIC: The subject stands under the gantry, holding a steel wire to perform forward and side bending, rotating the upper body about 45° while generating the maximum force, with the tester applying a counterforce from the side rear, holding for 3–5 s, collecting electromyographic data of the external oblique abdominal muscle (Saeterbakken et al., 2019).
(3) Asymmetrical load bench press test: Subjects in the asymmetric load bench press test adhered to the standard bench press movement, executing bench press offset training under four distinct asymmetric load interventions: 0%, 2%, 4%, and 6%. Warm-up routines preceding the bench press training and movement standards during the training session were consistent with prior descriptions. The 8-channel Noraxon wireless surface electromyographic signal acquisition device was employed to measure and analyze the bioelectric potential of muscles throughout each asymmetric bench press offset training session. Concurrently, the Enode pro sports performance strength and power collection device was utilized to record the movement speed and power of the barbell rod. The entire test procedure was video-synchronized, capturing the surface electromyographic signals and bench press performance of various muscles during different degrees of asymmetric load bench press offset training. Additionally, blood lactate and heart rate indicators of the subjects were collected both before and after the training (Figure 5). The EmgServer3.0 analysis software was employed to analyze the mean peak surface area electrical signal during the exhaustion phase of the asymmetric load bench press offset test for each group (specifically, the final four bench presses) (Krzysztofik et al., 2021). Raw electromyographic signals were refined using an 8–450 Hz bandpass filter (a Butterworth second-order bandpass filter) and full-wave rectification to derive the RMS for each target muscle throughout the bench press. For the MVIC test, electromyographic signals from 0.5 s before and after the maximum value were processed in the aforementioned manner to ascertain the RMS of each target muscle during the MVIC. The desired standardized RMS, denoted as MVIC%, was calculated by dividing the RMS from the bench press phase for each target muscle by the RMS from the MVIC test. This metric indicates the activation level of the target muscles. To determine the MDF index value, the time-domain signal was transformed into a frequency-domain signal *via* fast Fourier transform (FFT). The power of each frequency component was then computed. The resulting power spectrum was aggregated to derive the cumulative power distribution, with the MDF representing the frequency value that accounts for 50% of the total power in this distribution.

(4) Experimental Control: 1. Timing Control: To ensure that differences in training times do not affect the results, all subjects will complete each experimental test during the same morning timeframe, from 8:30 to 11:30. This approach minimizes the impact of circadian rhythms on the findings. Additionally, a 48-h washout period is implemented between the asymmetric load bench press training sessions of varying degrees to eliminate cumulative effects, thereby ensuring the objectivity of the experimental outcomes.2. Physical Activity and Dietary Control: Throughout the washout period, participants were barred from engaging in additional physical exercise to mitigate the impact of extraneous physical activity on athletic performance and minimize the risk of sports injuries. This measure ensured reduced inconsistencies in experimental conditions among individuals. Furthermore, the experiment was structured as a self-controlled crossover acute study, necessitating each participant’s attendance five times—once for a bench press 1 R M test and four times for varying degrees of asymmetric load bench press offset training tests. Consequently, stringent records of daily intake of three meals were maintained by test personnel throughout the entire experimental duration to ensure consistency in dietary intake among all subjects. Additionally, participants were imperatively required to abstain from consuming any drugs or sports supplements that could potentially enhance athletic performance during the experimental period, thereby minimizing the risk of additional variables influencing athletic performance.

### 2.3 Statistical analysis

The data were statistically analyzed using SPSS 26.0 software and graphed using Graphpad Prism 8.0. The original data are expressed in the form of mean ± standard deviation (M±SD). After the homogeneity of variance test, for univariate analysis, independent sample t-tests were used for comparisons between two groups, and paired sample t-tests were used for within-group comparisons; one-way repeated measures ANOVA was used for multiple group comparisons. The statistical significance level was set at p < 0.05; a very significant level was p < 0.01.

## 3 Results

### 3.1 Raw EMG of the target muscles when subjects performed the asymmetrical load bench press offset training

**FIGURE 6:**
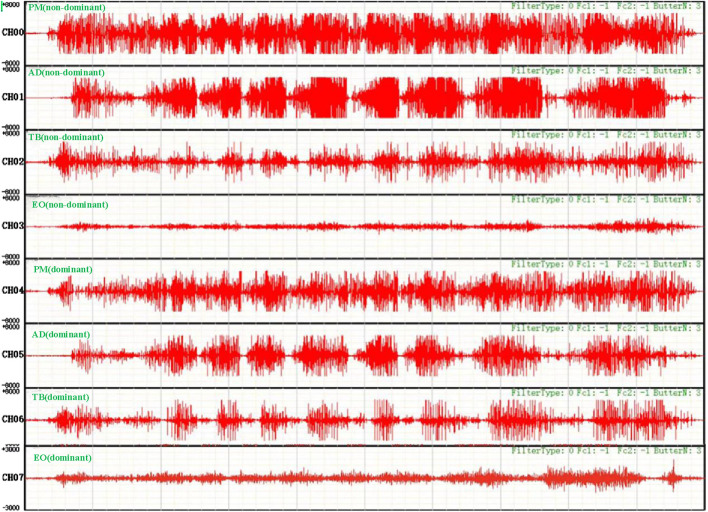
Original EMG of a target muscle during an asymmetric load bench press offset training of a subject.

Specific experimental results are shown in Figure 6.

#### 3.1.1 Variations in the MVIC% values of the targeted muscle groups during asymmetric load bench press offset training

**FIGURE 7:**
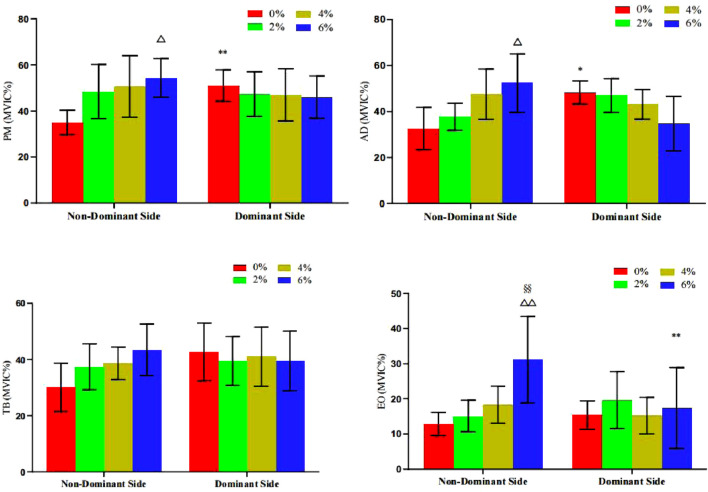
The change of MVIC% value of target muscle group during bench press offset training with different asymmetric load.

The results in Table 2 and Figure 7 indicate that bilateral muscle group comparisons within the group revealed the following: The MVIC% values of the non-dominant side pectoralis major (P = 0.009) and anterior deltoid (P = 0.038) were significantly lower than those on the dominant side when training with 0% asymmetry. With 6% asymmetry, the MVIC% value of the non-dominant side external oblique muscle was significantly higher than the dominant side (P = 0.004), while no significant differences were observed in other groups. When comparing intervention groups for the same muscle group asymmetry, it was found that the MVIC% values of the non-dominant side pectoralis major (P = 0.035) and anterior deltoid (P = 0.022) at 6% asymmetry were both significantly higher than those of the 0% load group; moreover, the activation level of the non-dominant side external oblique muscle was not only significantly higher than the 0% load group (P < 0.001), but also significantly better than the 2% load group (P = 0.005), with no significant differences between other groups.

**TABLE 2:** Summary of changes in MVIC% values of target muscle groups during bench press offset training with different asymmetric loads (n = 10).

Muscle group	Asymmetric intervention	Non-dominant side	Dominant side	F	η^2^
Pectoralis major (PM)	0% asymmetric	35 ± 5.32	51 ± 6.82**	0.195	0.005
	2% asymmetric	48.4 ± 11.8	47.4 ± 9.71		
	4% asymmetric	50.7 ± 13.3	47 ± 11.4		
	6% asymmetric	54.4 ± 8.46^△^	46 ± 9.17		
Anterior deltoid (AD)	0% asymmetric	32.6 ± 9.21	48.2 ± 5.05*	0.169	0.005
	2% asymmetric	37.6 ± 5.88	47 ± 7.33		
	4% asymmetric	47.5 ± 10.9	43.1 ± 6.48		
	6% asymmetric	52.3 ± 12.7^△^	34.8 ± 11.9		
Triceps brachii (TB)	0% asymmetric	30.2 ± 8.63	42.7 ± 10.3	2.84	0.073
	2% asymmetric	37.4 ± 8.19	39.5 ± 8.66		
	4% asymmetric	38.7 ± 5.84	41 ± 10.5		
	6% asymmetric	43.5 ± 9.20	39.6 ± 10.6		
External oblique (EO)	0% asymmetric	12.8 ± 3.34	15.4 ± 4.06	3.19	0.081
	2% asymmetric	15.1 ± 4.47	19.6 ± 8.06		
	4% asymmetric	18.4 ± 5.29	15.2 ± 5.17		
	6% asymmetric	31.1 ± 12.3^△△§§^	17.4 ± 11.5**		

Note: * indicates a significant difference in muscle MVIC% value changes between the non-dominant and dominant sides of the same muscle group within the experimental group (*.p < 0.05; **.p < 0.01); under the same muscle group, △ indicates a significant difference in muscle group MVIC% value changes compared to 0% asymmetry intervention (△.p < 0.05; △△.p < 0.01), § indicates a significant difference in muscle group MVIC% value changes compared to 2% asymmetry intervention (§.p < 0.05; §§.p < 0.01), and # indicates a significant difference in muscle group MVIC% value changes compared to 4% asymmetry intervention (#.p < 0.05; ##.p < 0.01).

### 3.2 Changes in the MDF values of the target muscle groups in different asymmetric load bench press offset training

**FIGURE 8:**
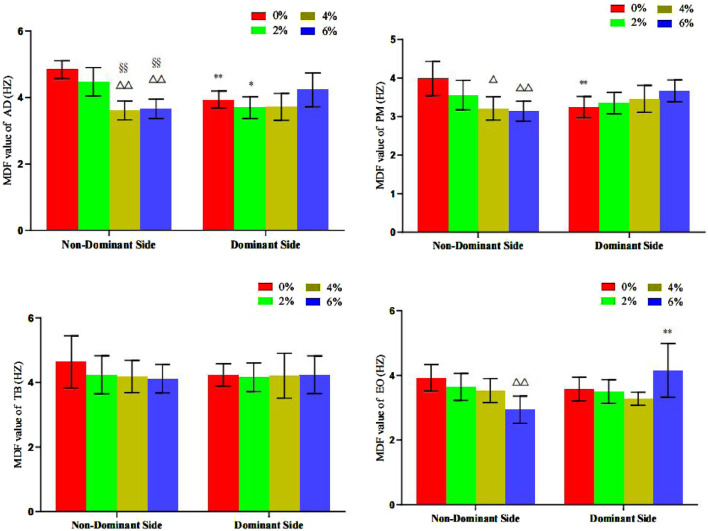
The change of MDF value of target muscle group during bench press offset training with different asymmetric load.

The findings from Table 3 and Figure 8 indicate that in within-group bilateral muscle group comparisons, the mean difference force (MDF) values for the non-dominant pectoralis major (P = 0.002) and anterior deltoid (P = 0.002) at 0% asymmetry were notably higher on the non-dominant side during asymmetrical bench pressing. The MDF value of the non-dominant anterior deltoid at 2% asymmetry was significantly elevated compared to the dominant side (P = 0.017). Conversely, the MDF value of the non-dominant external oblique muscles at 6% asymmetry was substantially reduced in comparison to the dominant side (P < 0.001). No significant disparities were observed in other groups. When comparing intervention groups with identical muscle group asymmetry, it was determined that: the MDF values of the pectoralis major at 4% (P = 0.01) and 6% asymmetry (P = 0.002) were both significantly diminished relative to the 0% load group; the MDF values of the anterior deltoid at 4% (P < 0.001) and 6% asymmetry (P < 0.001) were not only significantly reduced compared to the 0% load group but also significantly lower than the 2% asymmetry (P = 0.001); furthermore, the MDF value of the external oblique muscles at 6% asymmetry was significantly decreased compared to the 0% load group (P = 0.002), with no significant differences noted between other groups.

**TABLE 3:** Summary of changes in MDF values of target muscle groups during bench press offset training with different asymmetric loads (n = 10).

Muscle group	Asymmetric intervention	Non-dominant side	Dominant side	F	η^2^
Pectoralis major (PM)	0% asymmetric	3.99 ± 0.45	3.25 ± 0.27**	0.365	0.010
	2% asymmetric	3.56 ± 0.38	3.35 ± 0.28		
	4% asymmetric	3.21 ± 0.30^△^	3.46 ± 0.35		
	6% asymmetric	3.14 ± 0.26^△△^	3.67 ± 0.29		
Anterior deltoid (AD)	0% asymmetric	4.84 ± 0.27	3.94 ± 0.26**	9.27	0.205
	2% asymmetric	4.47 ± 0.42	3.69 ± 0.33*		
	4% asymmetric	3.61 ± 0.28^△△§§^	3.72 ± 0.41		
	6% asymmetric	3.66 ± 0.29^△△§§^	4.23 ± 0.51		
Triceps brachii (TB)	0% asymmetric	4.63 ± 0.81	4.23 ± 0.35	0.476	0.013
	2% asymmetric	4.23 ± 0.59	4.16 ± 0.44		
	4% asymmetric	4.18 ± 0.50	4.21 ± 0.70		
	6% asymmetric	4.12 ± 0.45	4.24 ± 0.59		
External oblique (EO)	0% asymmetric	3.93 ± 0.41	3.58 ± 0.37	1.23	0.033
	2% asymmetric	3.64 ± 0.41	3.5 ± 0.37		
	4% asymmetric	3.53 ± 0.35	3.28 ± 0.20		
	6% asymmetric	2.94 ± 0.28^△△^	4.16 ± 0.83**		

Note: * indicates a significant difference in muscle MDF, values between the non-dominant and dominant sides of the same muscle group within the experimental group (*.p < 0.05; **.p < 0.01); under the same muscle group, △ indicates a significant difference in muscle MDF, values compared to 0% asymmetric intervention (△.p < 0.05; △△.p < 0.01), § indicates a significant difference in muscle MDF, values compared to 2% asymmetric intervention (§.p < 0.05; §§.p < 0.01), and # indicates a significant difference in muscle MDF, values compared to 4% asymmetric intervention (#.p < 0.05; ##.p < 0.01).

### 3.3 Variations in bench press movement performance-related indicators under different asymmetrical load bench press offset trainings

#### 3.3.1 Variations in the movement speed of the barbell

**FIGURE 9:**
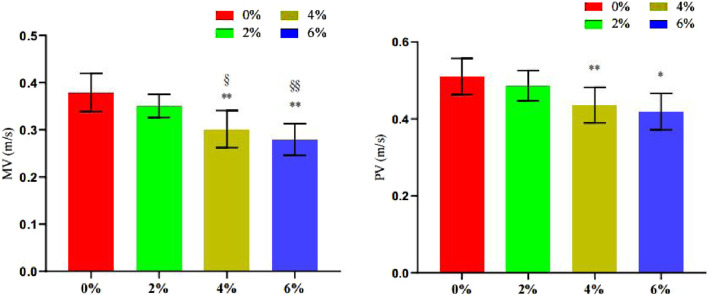
The change of barbell speed during bench press offset training with different asymmetrical loads.

The findings from Table 4 and Figure 9 indicate that the mean velocity (MV) values for both 4% asymmetry (P = 0.002) and 6% asymmetry (P < 0.001) were significantly lower than that of 0% asymmetry during the asymmetrical bench press. Furthermore, the MV values for these two groups were also significantly lower than that of 2% asymmetry (4% asymmetry P = 0.019, 6% asymmetry P < 0.001). The peak velocity (PV) values for 4% asymmetry (P = 0.011) and 6% asymmetry (P = 0.002) were significantly lower than that of 0% asymmetry, with the PV value for 6% asymmetry also significantly lower than that of 2% asymmetry (P = 0.014). No significant differences were observed in the other groups.

**TABLE 4:** Summary of changes in barbell movement speed indexes in bench press offset training with different asymmetric loads (n = 10).

	0% asym	2% asym	4% asym	6% asym	F
MV	0.38 ± 0.04	0.35 ± 0.02	0.30 ± 0.04**^§^	0.28 ± 0.03**^§§^	15.56
PV	0.51 ± 0.05	0.49 ± 0.04	0.44 ± 0.05**	0.42 ± 0.05*	8.00

Note: * indicates a significant difference in the change of index values compared to the 0% asymmetric intervention group (*.p < 0.05; **.p < 0.01), and § indicates a significant difference in the change of index values compared to the 2% asymmetric intervention group (§.p < 0.05; §§.p < 0.01).

#### 3.3.2 Changes in the power of barbell movement

**FIGURE 10:**
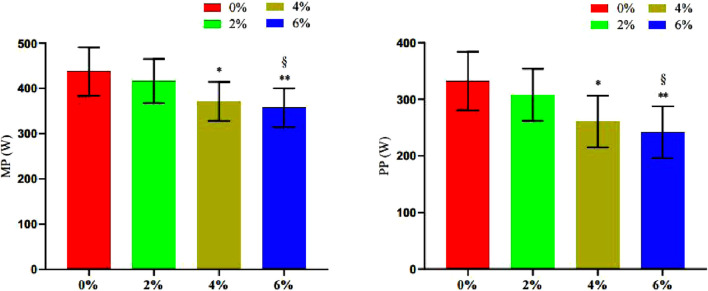
Changes in the number of bench press repetitions and time under tension during different asymmetric load shift training.

The findings presented in Table 5 and Figure 10 indicate that the Mean Pairwise (MP) values for 4% asymmetry (P = 0.034) and 6% asymmetry (P = 0.009) are significantly lower than that of 0% asymmetry. Furthermore, the MP value for 6% asymmetry is significantly lower than that of 2% asymmetry (P = 0.046). The Pairwise Proportion (PP) values for 4% asymmetry (P = 0.02) and 6% asymmetry (P = 0.003) are also significantly lower than that of 0% asymmetry. Additionally, the PP value for 6% asymmetry is significantly lower than that of 2% asymmetry (P = 0.023). No significant differences were observed in the other groups.

**TABLE 5:** Summary of changes in barbell motion power index in bench press offset training with different asymmetric loads (n = 10).

	0% asym	2% asym	4% asym	6% asym	F
MP	437 ± 53.70	416 ± 48.70	371 ± 43*	357 ± 43**^§^	7.01
PP	332 ± 51.60	308 ± 46.10	261 ± 45.80*	242 ± 45.70**^§^	5.73

Note: * indicates a significant difference in the change of index values compared to the 0% asymmetric intervention group (*.p < 0.05; **.p < 0.01), and § indicates a significant difference in the change of index values compared to the 2% asymmetric intervention group (§.p < 0.05; §§.p < 0.01).

#### 3.3.3 Changes in the number of repetitions and duration under tension in the bench press exercise

**FIGURE 11:**
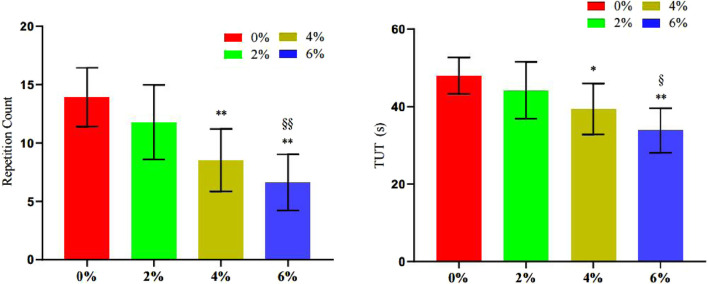
Changes in the number of bench press repetitions and time under tension during different asymmetric load shift training.

The findings presented in Table 6 and Figure 11 indicate that the repetition times values for 4% asymmetry (P = 0.001) and 6% asymmetry (P < 0.001) were significantly lower than those for 0% asymmetry. Furthermore, the repetition times value for 6% asymmetry was notably lower than that for 2% asymmetry (P = 0.004). Similarly, the TUT values for 4% asymmetry (P = 0.018) and 6% asymmetry (P < 0.001) were significantly lower than that of 0% asymmetry. The TUT value for 6% asymmetry was also significantly lower than that for 2% asymmetry (P = 0.013). No significant differences were observed in the other groups.

**TABLE 6:** Summary of changes in bench press repetitions and time under tension in different asymmetric load offset training (n = 10).

	0% asym	2% asym	4% asym	6% asym	F
Number of repetitions	13.90 ± 2.52	11.80 ± 3.19	8.53 ± 2.67 **	6.63 ± 2.40**^§§^	15.65
TUT	48 ± 4.71	44.20 ± 7.35	39.40 ± 6.55*	33.90 ± 5.72**^§^	12.12

Note: * indicates a significant difference in the change of index values compared to the 0% asymmetric intervention group (*.p < 0.05; **.p < 0.01), and § indicates a significant difference in the change of index values compared to the 2% asymmetric intervention group (§.p < 0.05; §§.p < 0.01).

### 3.4 Changes in physiological indicators before and after bench press training with different asymmetrical loads

#### 3.4.1 Changes in blood lactate

**FIGURE 12:**
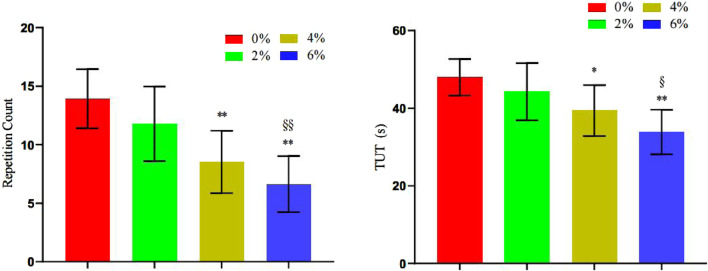
Changes in the number of bench press repetitions and time under tension during different asymmetric load shift training.

The findings from Table 7 and Figure 12 reveal that the blood lactate values for both the 4% asymmetry (P = 0.003) and 6% asymmetry (P = 0.003) groups post-asymmetrical bench press were notably lower than those of the 0% asymmetry group after the same exercise. Furthermore, the blood lactate levels for the 4% asymmetry (P = 0.036) and 6% asymmetry (P = 0.037) groups post-exercise were significantly reduced compared to the 2% asymmetry group’s post-exercise values. No significant differences were observed in the other groups.

**TABLE 7:** Summary of changes in blood lactic acid before and after bench press offset training with different asymmetric loads (n = 10).

	0% asym	2% asym	4% asym	6% asym	F
Pre-test	1.83 ± 0.24	1.8 ± 0.31	1.78 ± 0.31	1.68 ± 0.32	0.459
Post-test	9.88 ± 0.75	9.14 ± 0.71	7.43 ± 1.57**^§^	7.42 ± 1.59**^§^	10.406

Note: * indicates a significant difference in the change of index values compared to the 0% asymmetric intervention group (*.p < 0.05; **.p < 0.01), and § indicates a significant difference in the change of index values compared to the 2% asymmetric intervention group (§.p < 0.05; §§.p < 0.01).

#### 3.4.2 Changes in heart rate

**FIGURE 13:**
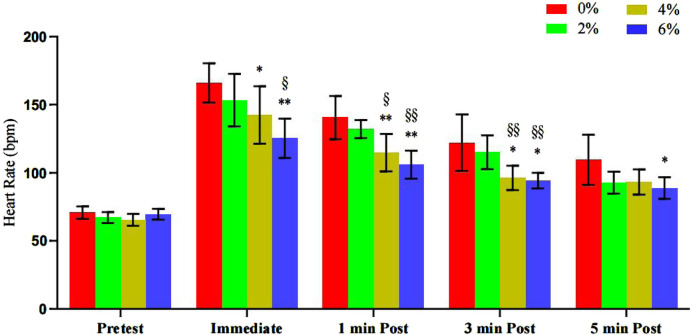
Changes before and after bench press offset training with different asymmetric loads.

The results of Table 8 and Figure 13 showed that compared with 0% asymmetry, the HR values were significantly increased at 0 min 4% asymmetry (P = 0.047) and 6% asymmetry (P < 0.001); 1 min 4% asymmetry (P = 0.006) and 6% asymmetry (P < 0.001); 3 min 4% asymmetry (P = 0.016) and 6% asymmetry (P = 0.01); and 5 min 6% asymmetry (P = 0.029). Compared with 2% asymmetry, the HR values were significantly increased at 0 min 6% asymmetry (P = 0.01); 1 min 4% asymmetry (P = 0.0015) and 6% asymmetry (P < 0.001); 3 min 4% asymmetry (P = 0.006) and 6% asymmetry (P = 0.002), and no significant differences were found in the other groups.

**TABLE 8:** Summary of heart rate changes before and after bench press offset training with different asymmetric loads (n = 10).

HR	0% asym	2% asym	4% asym	6% asym	F
Pre-test	70.6 ± 4.58	67 ± 4	65.3 ± 4.39	69.4 ± 3.99	2.76
0min	166 ± 14.5	153 ± 19.3	142 ± 21.1*	125 ± 14.4**^§^	12.85
1min	140 ± 15.8	132 ± 6.57	115 ± 13.7**^§^	106 ± 10.2**^§§^	19.04
3min	122 ± 20.8	115 ± 12.4	96.2 ± 8.91*^§§^	94.3 ± 5.69*^§§^	11.42
5min	109 ± 18.5	92.6 ± 8.13	93.1 ± 9.43	88.7 ± 8.09*	3.34

Note: * indicates a significant difference in the change of index values compared to the 0% asymmetric intervention group (*.p < 0.05; **.p < 0.01), and § indicates a significant difference in the change of index values compared to the 2% asymmetric intervention group (§.p < 0.05; §§.p < 0.01).

## 4 Discussion

### 4.1 Effects of symmetric load bench press training on muscle activation and exercise-induced fatigue

Inter-limb asymmetry serves as the theoretical foundation for offset training. Extensive research has demonstrated that inter-limb asymmetry, characterized by significant disparities in muscle activation between bilateral limbs, occurs not only in elite weightlifters (Golas et al., 2018) but also in general fitness populations (Krzysztofik et al., 2021). However, whether bodybuilders exhibit such asymmetry remains inconclusive. This study simulated symmetric load bench press training (0% asymmetry) to investigate its effects on muscle activation and exercise-induced fatigue in bodybuilders. The results revealed significant inter-limb asymmetry during symmetric loading (70% 1 R M), with the dominant-side pectoralis major (PM: 51 ± 6.82 vs 35 ± 5.32, p = 0.009) and anterior deltoid (AD: 48.2 ± 5.05 vs 32.6 ± 9.21, p = 0.038) exhibiting higher MVIC% values than the non-dominant side. These findings align with studies by Golas et al. (2018) and Krzysztofik et al. (2021). For instance, Golas et al. (2018) observed that elite powerlifters under 70% and 90% 1 R M loads demonstrated significantly higher total electromyographic (EMG) peaks on the dominant side, particularly exceeding 100% MVIC for the anterior deltoid and triceps brachii at 90% 1 R M. Similarly, resistance-trained individuals with over 6 years of experience showed dominant-side dominance in anterior deltoid and triceps activation across low (50% 1 R M) and high (90% 1 R M) loads (p < 0.001), though no significant differences were observed in PM activation (p = 0.168) (Krzysztofik et al., 2021). Collectively, these results underscore the prevalence of inter-limb asymmetry across populations, reinforcing the necessity of asymmetric load interventions to mitigate long-term performance deficits and injury risks.

Notably, unlike previous studies emphasizing triceps asymmetry, this study identified PM and AD as the primary asymmetrical muscles in bodybuilders. This discrepancy may stem from training specialization: Bodybuilders prioritize targeted muscle recruitment over maximal load lifting. Despite the bench press engaging PM, AD, and triceps brachii (TB), PM contributes most significantly to the movement (Padulo et al., 2015), making its asymmetry more pronounced in this population. To evaluate exercise-induced fatigue, median frequency (MDF), blood lactate, and heart rate were systematically analyzed. MDF reflects muscle fatigue, with declining values indicating increased low-frequency EMG components due to metabolic byproduct accumulation and impaired neuromuscular conduction (Daniel and Małachowski, 2023). Dominant-side PM and AD exhibited lower MDF values, suggesting higher fatigue levels. Post-exercise blood lactate increased from 1.83 ± 0.24 mmol/L to 9.88 ± 0.75 mmol/L, and heart rate rose from 70.6 ± 4.58 bpm to 166 ± 14.5 bpm, though trends lacked statistical significance. Elevated lactate correlates with glycolytic metabolism during high-intensity exercise, exceeding lactate clearance thresholds and inducing central fatigue (Hideaki and Yusuke, 2013). Heart rate modulation, governed by autonomic nervous system activity, aligns with sympathetic activation during exertion and parasympathetic recovery post-exercise (Marasingha-Arachchige et al., 2020). Overall, non-dominant muscles exhibited greater fatigue, yet symmetric loading did not induce significant exercise-induced fatigue.

### 4.2 Effects of graded asymmetric load bench press offset training on muscle activation and fatigue

Current research focuses on identifying optimal asymmetric load thresholds to enhance non-dominant muscle activation while minimizing compensatory fatigue. Jarosz et al. (2020) demonstrated that acute asymmetric loading (0%–7.5% 1 R M) at 70% 1 R M significantly improved non-dominant PM and AD activation, particularly at 7.5%. Conversely, Saeterbakken et al. (2020b) observed that higher asymmetric loads (5%–10% 1 R M) reduced non-dominant PM and AD activation but increased external oblique (EO) activity by 280%–320%, indicating core compensation. In this study, 0%–6% asymmetric loads were applied, with non-dominant loading and dominant unloading to maintain total load at 70% 1 R M. Moderate interventions (2%–4%) effectively reduced asymmetry by elevating non-dominant PM and AD activation (6% PM: 54.4% ± 8.46% vs 0%: 35 ± 5.32, p = 0.035), consistent with Jarosz et al. (2020). However, activation plateaued at higher loads (6%), suggesting a 4% threshold for maximizing non-dominant recruitment. Excessive loading (6%) triggered core compensation (non-dominant EO: 31.1% ± 12.3% vs 0%: 12.8 ± 3.34, p < 0.001), diverting effort from primary movers and contradicting training objectives.

Performance metrics (barbell velocity, power, repetitions, time under tension) and physiological markers (blood lactate, heart rate) deteriorated progressively with higher asymmetry. For example, 6% loading reduced mean velocity (MV: 0.28% ± 0.03% vs 0%: 0.38 ± 0.04, p < 0.001) and increased post-exercise lactate (7.42% ± 1.59% vs 0%: 9.88 ± 0.75, p = 0.003). These findings align with Matthew Sharp et al. (2022), where 5% asymmetric loading enhanced hypertrophy and strength but increased perceived exertion, highlighting trade-offs between efficacy and fatigue.

Recent evidence has shed light on the neural control mechanisms underlying limb dominance, which is highly relevant to understanding the enhanced non-dominant muscle activation observed in our study. Lecce et al. (2025) demonstrated that dominant muscles exhibit greater MVF compared to non-dominant muscles, associated with higher motor unit discharge rates and a greater proportion of common synaptic inputs. This suggests that the higher strength and activation levels in dominant limbs are primarily driven by increased neural drive due to greater shared synaptic inputs rather than differences in intrinsic motoneuron properties. Similarly, our study found that non-dominant muscles under asymmetric loading showed increased activation, which may be attributed to altered neural drive mechanisms. The application of asymmetric loads could potentially modulate the distribution of synaptic inputs, enhancing the neural drive to the non-dominant limb and thereby improving its activation levels.

Moreover, the phenomenon of cross-education, where unilateral training induces adaptations in the contralateral untrained limb, has been increasingly explored. Lecce et al. (2024) highlighted that unilateral resistance training can lead to significant strength gains in the non-trained contralateral limb, mediated by motor unit adaptations. This cross-education effect may be explained by neural adaptations occurring at both spinal and supraspinal levels, including enhanced interhemispheric communication and reduced inhibitory mechanisms. In the context of asymmetric load training, the increased activation of non-dominant muscles might be influenced by similar cross-education mechanisms. The neural drive to the non-dominant limb could be potentiated through the coordinated activation of neural pathways, even in the absence of direct physical training, leading to improved muscle activation and force generation.

These neural adaptations may also play a role in the observed performance metrics and physiological fatigue markers in our study. The progressive decline in barbell velocity, power output, and repetitions with higher asymmetry levels could be partly due to the neural challenges of maintaining stability and coordination under asymmetric loading. Additionally, the increased blood lactate and prolonged heart rate recovery in the 6% asymmetry group suggest that the enhanced neural drive to muscles comes at the cost of increased metabolic and cardiovascular stress. This aligns with the notion that greater neural activation can lead to higher energy demands and faster fatigue onset.

In conclusion, the integration of recent findings on neural control mechanisms and cross-education provides a more comprehensive understanding of the enhanced non-dominant muscle activation and the associated physiological responses observed during asymmetric load bench press training. These insights not only enrich the interpretation of our results but also highlight the potential for targeted neural adaptations through asymmetric training protocols, which could be further explored in future research.

### 4.3 Study limitations

This study has several limitations.

(1) The acute experimental design involved short-term interventions of asymmetrical load bench press training with lateral displacement, which may not fully capture long-term adaptive changes. Chronic training could induce structural muscle remodeling, enhanced neuromuscular control, and sustained performance improvements—effects unobservable in acute experiments. Future studies should adopt longitudinal intervention designs to track participants’ adaptations over weeks or months, thereby comprehensively evaluating the long-term impacts of asymmetrical load training.
(2) The sample comprised only 10 collegiate bodybuilders, a limited and specific population, which restricts the generalizability of findings. Responses to asymmetrical load training may vary across athletes of different ages, training levels, and genders. Expanding the sample size to include participants with diverse backgrounds and training experiences would enhance the representativeness and external validity of results.
(3) Exercise-induced fatigue was primarily assessed using median frequency (MDF), blood lactate, and heart rate. These metrics may inadequately reflect the multidimensional complexity of fatigue. Future research should incorporate additional indicators, such as muscle strength/endurance tests, neuromuscular control assessments, psychological fatigue questionnaires, creatine kinase (to evaluate skeletal muscle damage), and oxidative stress markers (e.g., malondialdehyde [MDA] and superoxide dismutase [SOD]), to holistically assess the effects of asymmetrical load training on fatigue.

### 4.4 Innovations and contributions

This study revealed significantly higher activation levels in the dominant-side pectoralis major and anterior deltoid compared to the non-dominant side. In contrast to previous findings showing no significant pectoralis major asymmetry but notable differences in anterior deltoid and triceps activation, this discrepancy may stem from bodybuilders’ compensatory activation patterns due to prolonged symmetric bench press training. Regarding intervention efficacy, both 2% and 4% asymmetrical loads effectively reduced inter-limb asymmetry, with 2% demonstrating optimal training efficacy based on muscle activation and performance metrics. Notably, the 6% load partially mitigated asymmetry but yielded no significant additional benefits, aligning with Saeterbakken et al.‘s conclusion that excessive asymmetry triggers compensatory core muscle recruitment to maintain stability, contradicting the intervention’s original intent.

Key contributions include: (1) First systematic investigation of asymmetrical load bench press models in bodybuilders, uncovering latent inter-limb asymmetry under conventional symmetric training. (2) Empirical evidence for precision training protocols by quantifying how load gradients modulate target muscle activation. (3) Multidimensional insights (sport biomechanics, electromyographic, and physiological) advancing the understanding of neuromuscular coordination mechanisms during asymmetrical training. (4) Future research should integrate muscle function metrics, neuromuscular control parameters, biochemical markers, and psychophysiological indices to comprehensively evaluate exercise-induced fatigue.

## 5 Conclusion

This study explored the effects of graded asymmetrical load bench press training on muscle activation and exercise-induced fatigue in bodybuilders. Key findings include:

Inter-limb asymmetry exists in bench press training, with significantly lower activation levels in non-dominant pectoralis major and anterior deltoid.

2%, 4%, and 6% asymmetrical loads ameliorated asymmetry and enhanced non-dominant muscle activation. The 2% load demonstrated optimal efficacy with minimal performance impairment and fatigue.

Excessive asymmetry (6%) induced core muscle compensation and pronounced fatigue, detrimentally affecting performance. These findings highlight the importance of load optimization in asymmetrical training protocols to balance efficacy and physiological strain.

## Data Availability

The raw data supporting the conclusions of this article will be made available by the authors, without undue reservation.
