# Supplementary material for: Effects of asymmetric load bench press offset training on muscle activation levels and exercise-induced fatigue in collegiate bodybuilders
Source: Front Physiol. 2025 May 9;16:1592477. doi: 10.3389/fphys.2025.1592477 (PMC12098615; doi:10.3389/fphys.2025.1592477)
Supplement: Supplementary file 1 [file Table1.doc]

Informed Consent Form

1、 Research objective

The aim of this study is to investigate the effects of asymmetric load bench press offset training (unilateral load offset of 0%, 2%, 4%, 6% 1RM) on muscle activation levels (pectoralis major, anterior deltoid, triceps, external oblique muscle) and exercise fatigue (blood lactate, heart rate recovery, surface electromyographic frequency domain indicators) in college bodybuilders, providing scientific basis for optimizing strength training programs.

2、 Participation Content

Experimental procedure:

First test: Basic 1RM bench press assessment, body composition analysis (InBody 770).

Test 2-5: Complete bench press movements under 4 types of asymmetric loads (0%, 2%, 4%, 6% 1RM), with a training interval of ≥ 48 hours between each session.

Synchronize collection during each training session:

Surface electromyography (sEMG): RMS and MDF values of bilateral target muscles.

Blood lactate: Fingertip blood collection immediately before and after training (portable lactate analyzer).

Kinematic data: barbell trajectory and velocity (Vicon infrared motion capture system).

Time requirement: Approximately 4 weeks in total, with a single experiment lasting ≤ 90 minutes.

3、 Potential risks and discomfort

Muscle soreness or mild fatigue (risk controlled through 24-hour recovery monitoring and training load grading).

Fingertip blood collection may cause a brief stinging sensation (using disposable sterile blood collection needles, operated by licensed medical personnel).

4、 Benefit explanation

Free access to personalized muscle activation analysis reports (including sEMG data and training recommendations).

Prioritize participation in subsequent sports performance optimization courses.

5、 Confidentiality clause

All data is stored anonymously encoded (such as S001), and only the research team has access to the raw data.

The published results do not contain personally identifiable information.

6、 Voluntary participation and withdrawal rights

You have the right to withdraw unconditionally at any stage of the experiment without providing a reason.

After exiting, the collected data will be immediately destroyed (if you request).

7、 Informed confirmation

I have read and understood the above content, and voluntarily participate in this study.

Subject Signature: ________________ Date:_________

Researcher Signature: ________________ Date:_________
